# No difference in surgical time and total theatre time between robotically assisted and computer assisted total knee arthroplasty

**DOI:** 10.1186/s10195-024-00798-8

**Published:** 2024-11-08

**Authors:** David Johannes Haslhofer, Victoria Anelli-Monti, Peter Hausbrandt, Christian Kammerlander, Antonio Klasan

**Affiliations:** 1 Department for Orthopedics and Traumatology, Kepler University Hospital GmbH, Krankenhausstrasse 9, 4020 Linz, Austria; 2 Department for Orthopedics and Traumatology, Johannes Kepler University Linz, Altenberger Strasse 69, 4040 Linz, Austria; 3 Department for Orthopedics and Traumatology, AUVA Graz, Göstinger Straße 24, 8020 Graz, Austria; 4 Faculty of Medicine, Johannes Kepler University Linz, Altenbergerstrasse 69, 4040 Linz, Austria

**Keywords:** TKA, RAS, CAS, Robotically assisted arthroplasty, Total knee arthroplasty, Total knee replacement

## Abstract

**Introduction:**

A number of studies have demonstrated a significant reduction of surgical time for robotically assisted surgery (RAS) total knee arthroplasty (TKA) after the learning curve between 6 and 43 cases. It is unknown if the logistics of RAS produce a longer total theatre time since published literature only reflects the surgical time. It is also unknown how RAS surgical and total theatre times compare with computer assisted surgery (CAS) TKA.

**Methods:**

This is a prospective study of 524 consecutive patients undergoing a CAS or a RAS TKA using the same cementless implant. We recorded age, sex, body mass index (BMI), incision time of the first case, total surgical time, total theatre time, length of stay and 90-day complication and readmission rate.

**Results:**

During the study period, 205 CAS and 199 RAS TKA were performed. There was no difference at baseline in age (*p* = 0.546), sex (*p* = 0.920) or BMI (*p* = 0.791). Surgical time for CAS was 78.3 (± 22.2) min and for RAS was 80.1 (± 25.7) min, *p* = 0.451. Total theatre time for CAS was 117.4 (± 27.8) min and 119.3 (± 30.7) min for RAS, *p* = 0.515. There was no difference in length of stay (*p* = 0.674), 90-day complication (*p* = 0.530) or readmission rate (*p* = 0.930). There was a difference in skin-incision average time for the first case (*p* = 0.022).

**Conclusions:**

Although theatre set-up for the first case is 5 min longer, RAS-TKA does not prolong the surgical time or total theatre time when compared with CAS-TKA. There was no reduction in case volume since the introduction of robotics.

**Level of evidence:**

III.

## Introduction

Knee arthroplasty is a critical orthopaedic procedure for patients suffering from debilitating knee joint conditions [1, 2]. In recent years, the field of knee arthroplasty has seen significant technological advancements with the introduction of robotically assisted surgery (RAS) built on the foundations set by computer assisted surgery (CAS) [3, 4].

These innovative approaches allowed a more personalized approach with more precision and accuracy of knee arthroplasty, leading to promising patient outcomes [3, 5–7], although reduced complications and enhanced implant longevity must be proven over the long-term [8]. However, the choice using RAS or CAS can substantially impact surgical time and total theatre time, making them pivotal factors in the preoperative planning and execution of these procedures [3].

A number of studies have demonstrated a significant reduction of surgical time for robotically assisted (RAS) total knee arthroplasty (TKA) after the learning curve between 6 and 43 cases [6, 9–11]. The learning curve has been demonstrated to be virtually non-existent in the presence of a RAS-experienced surgeon [12]. RAS however, involves different prepping and draping procedures which have not yet been reflected in literature.

The aim of this study is to evaluate the surgical and total theatre times of RAS and CAS as well as the comparison between these two different approaches.

It was hypothesized that RAS surgical and total theatre time is higher compared with CAS.

## Methods

### Ethics

The study was approved by the local regional ethical committee (ethics board approval xxx).

### Patients

Consecutive patients were included from a single centre prospectively collected database. In our centre approximately 400 primary and revision knee arthroplasties are performed per year. CAS (Orthomap, Stryker, Kalamazoo, MI, USA) has been the standard in the centre since 2012. RAS (MAKO, Stryker) was introduced in November 2021. Choice of technique was on the basis of the surgical slots available, with all surgeons except one performing both RAS and CAS.

Inclusion criteria were defined as TKA using CAS or RAS between 1 January 2022 and 30 September 2023, Fig. 1. We excluded the first 20 cases of each surgeon using RAS owing to the learning curve [12], except for the RAS only surgeon. We recorded age, sex and body mass index (BMI).

**Fig. 1:**
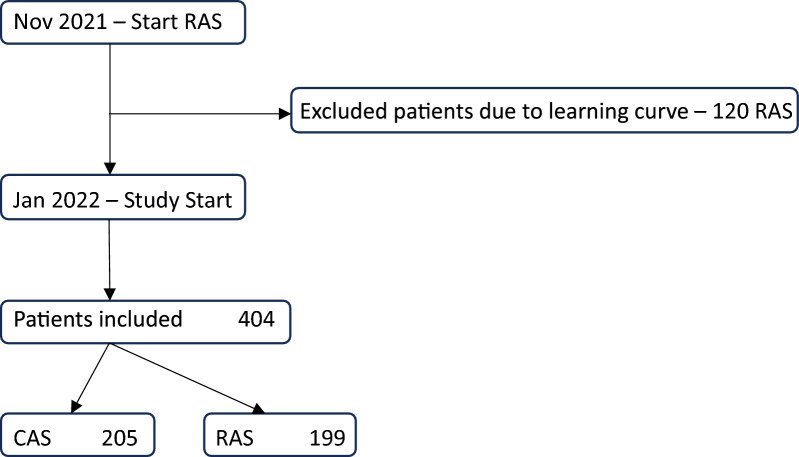
Patient flow chart

#### Outcome measures

The patients receive anaesthesia in an anaesthetic prep-room directly attached to the operating room, which was recorded by the anaesthetic nurse. The theatre nurse documents the beginning of the theatre time once opening of the sterile draping for the instrument tables began. This was followed by opening of the instruments and sterile draping of the MAKO (Stryker) system or, in case of CAS, setting up the CAS. After this stage, the patient is brought into theatre, prepped and draped. After team time out, the procedure begins, marking the beginning of the surgical time. Placing the last suture marks the end of the surgical time. Dressings are applied, an X-ray is performed and all instruments and disposables are removed. The patient leaves the theatre and once all instruments are removed and the cleaning staff can begin cleaning the room, the end of total theatre time is marked. The first patient of the day was brought to the anaesthetic room at 07:30:00.

In addition to surgical and theatre times, we recorded the length of stay and 90-day complication and readmission rates.

CAS was performed using mechanical alignment, whereas RAS was performed using either adjusted mechanical alignment (aMA) or functional alignment (FA) using previously described techniques [13, 14]. All patients received cementless TKA (Triathlon, Stryker). In total, seven surgeons performed or supervised all cases.

Complications were defined according to the classification by Clavien–Dindo [15].

### Statistics

Statistical analysis was performed using IBM SPSS Statistics 29 (Armonk, NY, USA). Data is reported using mean (± standard deviation, SD) for normally distributed data and median (interquartile range, IQR) for non-normally distributed data. Normally distributed data were compared using the paired *t*-test, non-normally distributed data using Mann–Whitney’s *U* test. Significance was set at *p* < 0.05. A post hoc power analysis has been performed as an equivalence trial. Using the SD of 20 min and an equivalence limit of 10 min, 69 patients per group would be required to demonstrate a difference.

## Results

During the study period 404 patients met the inclusion criteria – 205 CAS and 199 RAS primary TKA were performed. Age ranged from 38 to 94 years with a mean of 68.9 ± 11.5 years. There was no difference at baseline in age (CAS 67.5 ± 11.4 years versus RAS 68.8 ± 11.9 years, *p* = 0.546), sex (female CAS 49.8% versus RAS 50.2%, *p* = 0.920) or BMI (CAS 28.4 ± 8.1 versus RAS 28.2 ± 7.0 *p* = 0.791).

**Fig. 2:**
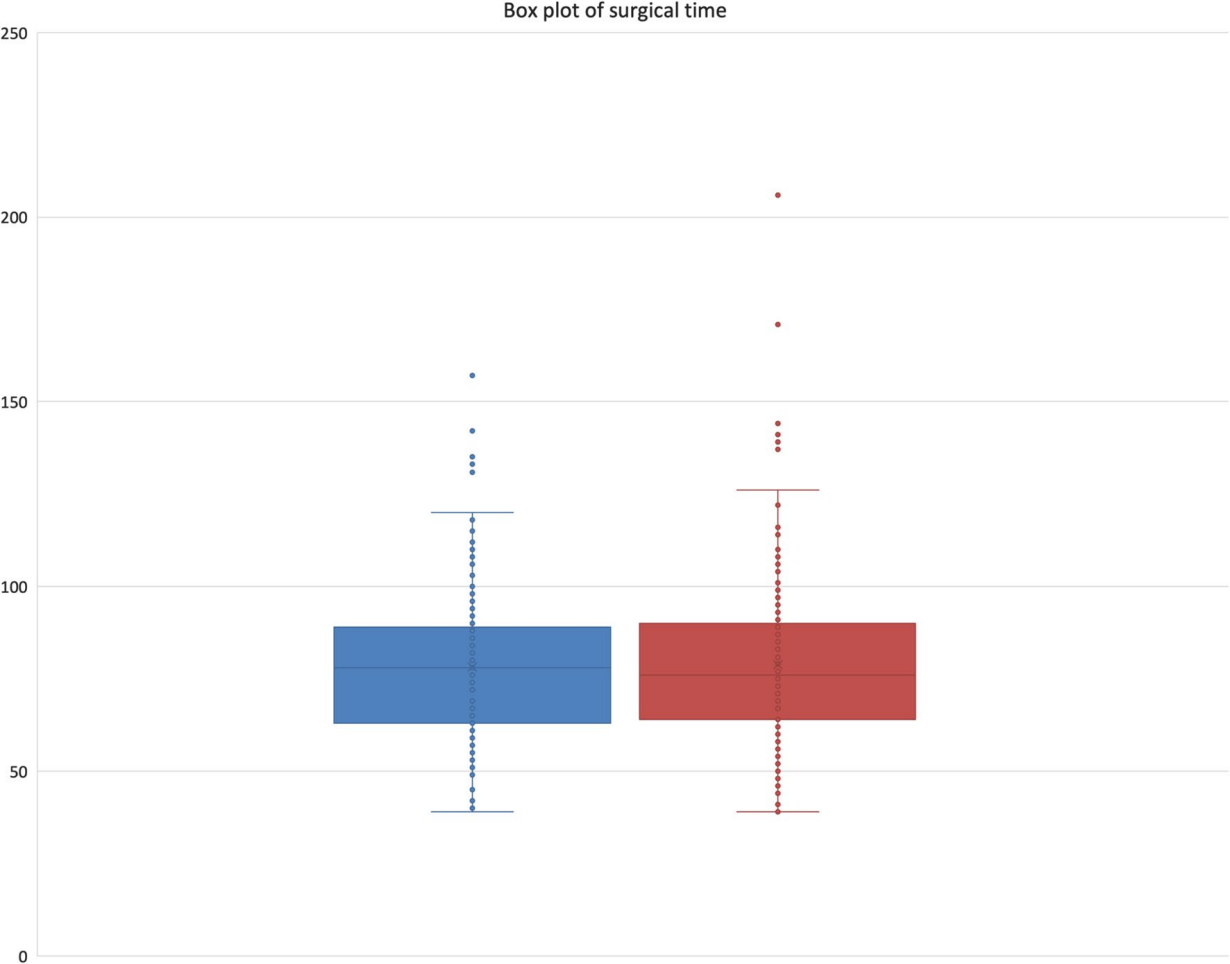
Surgical time, CAS is shown in blue and RAS in red

**Fig. 3:**
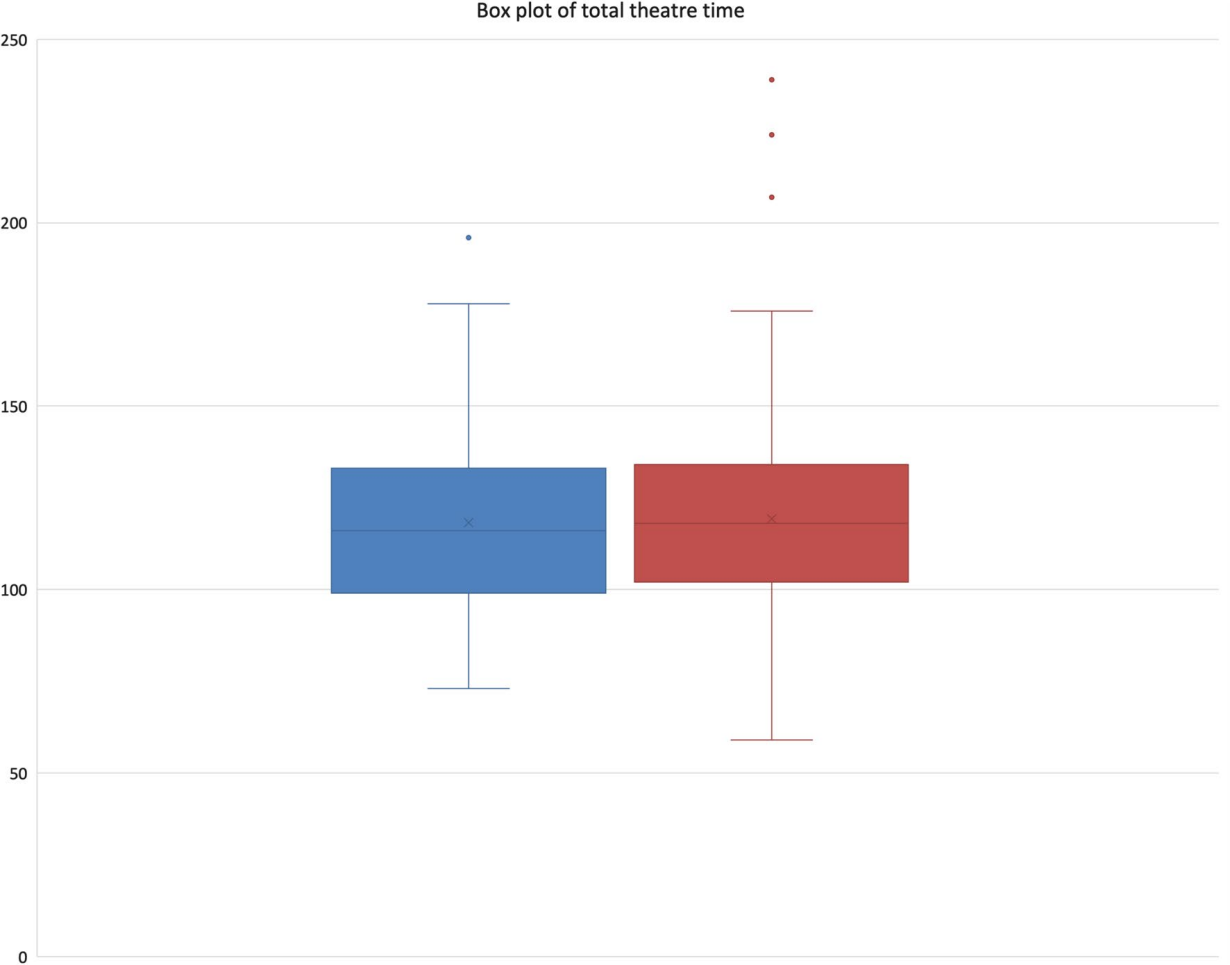
Total theatre time, CAS blue, RAS red

Surgical time for CAS was 78.3 (± 22.2) min and for RAS was 80.1 (± 25.7) min, *p* = 0.4513, Fig. 2. Total theatre time for CAS was 117.4 (± 27.8) min and 119.3 (± 30.7) min for RAS, *p* = 0.5145, Fig. 3. There was no difference in length of stay (CAS 4.7 ± 2.2 days versus RAS 4.6 ± 2.1 days, *p* = 0.674), ninety-day complication (CAS 5.4% versus RAS 3.9%, *p* = 0.530) or readmission rate (CAS 1.7% versus RAS 1.9%, *p* = 0.930). There was a statistically significant difference in skin-incision average time for the first case (08:29 AM for CAS versus 08:34 AM for RAS, *p* = 0.022) Table 1.

**Table 1:** Demographic data

	RAS	CAS
*n* Cases	199	205
Age (in years)	68.8 (± 11.9)	67.5 (± 11.4)
Female %	50.2	49.8
BMI	28.2 (± 7.0)	28.4 (± 8.1)
Surgical time (in minutes)	80.1 (± 25.7)	78.3 (± 22.2)
Total theatre time (in minutes)	119.3 (± 30.7)	117.4 (± 27.8)
90-day complication rate (in %)	3.9	5.4
Readmission rate (in %)	0.3	0.6

## Discussion

The most important finding of this study was that RAS-TKA does not prolong surgical or theatre time in comparison with CAS-TKA, refuting our hypothesis.

Earlier studies already showed that longer surgical time is a factor when comparing RAS-TKA and conventional TKA [8, 16, 17]. Similarly, studies have found CAS-TKA to be longer than conventional TKA [18–20]. Our study compared both RAS-TKA and CAS-TKA surgical times without finding any statistically significant difference, not matching with the data presented by Siddiqi et al. where RAS-TKA showed shorter operation time than CAS-TKA [21]. The difference might be a consequence of the fact that CAS has been the standard since 2012 at our institution. In our view, this fact was also a facilitator for the change towards RAS.

A more important point when speaking about CAS or RAS is the total theatre time, owing to the extra effort to prepare the needed machines and technology, which has largely been ignored in literature. Comparing conventional TKA and CAS/RAS-TKA, longer theatre times in CAS/RAS-TKA groups are usually described [22]. When comparing CAS-TKA and RAS-TKA in our study we could not find any statistically significant difference. The lack of a difference might be a consequence of the previous experience by the surgical staff since both technologies utilize optical trackers and a tower. One important difference between the two technologies, however, is the presence of a technician with the MAKO surgical system [23]. Currently, this is obligatory as per manufacturer and does facilitate both the implementation and the continuity of the workflow. As per latest data, this is the only system that requires a technician on site [24].

The safety data of the present study demonstrates no difference both in terms of complications and length of stay.

In 2021 Steffens et al. published on comparing costs of CAS-TKA and RAS-TKA where they found comparable in-hospital costs, but when including capital costs of surgical equipment and maintenance RAS-TKA showed statistically higher expenses [25]. Cotter et al. demonstrated that cost reduction for RAS is in fact by tray reduction, shorter in hospital stays and lower readmission rate [26]. This study demonstrates no loss of intraoperative time, typically estimated at 36 USD/min [27], which has not been demonstrated before.

There are a few limitations of this study that need to be acknowledged. Firstly, patients were randomly assigned to the two different surgery groups, but there was no controlled randomization, as only one of the surgeons performed RAS after its introduction. Furthermore, although data was collected through a prospective database, data observation was performed in a retrospective manner. Another limitation is the lack of clinical outcome data, which was never one of the parameters of our conducted study. The obligatory presence of a technician is only the case with RAS used in this study, so the data may not apply to other systems. Henceforth, other manufacturer data needs to be further investigated. The clinical settings and volumes of centres can vary greatly, and the results of our study might not apply to each centre. The different approaches to efficiency between surgeons, as well as experience, could affect the study results as well. The study therefore included as many cases as possible to try to represent a typical department in the country where the study was performed.

## Conclusions

Although theatre set-up for the first case is 5 min longer, RAS-TKA does not prolong the surgical time or total theatre time compared with CAS-TKA. Implementation of robotic assisted surgery did therefore not reduce the case volume.

## Data Availability

Not applicable.
